# Artificial Intelligence for Multimorbidity: Managing Complexity at Scale

**DOI:** 10.3389/ijph.2026.1608904

**Published:** 2026-01-29

**Authors:** Hajira Dambha-Miller, Zlatko Zlatev

**Affiliations:** 1 Primary Care Research Centre, University of Southampton, Southampton, United Kingdom; 2 Digital Health and Biomedical Engineering Research Group, School of Electronics and Computer Sciences, University of Southampton, Southampton, United Kingdom

**Keywords:** multimorbidities, artificial intelligence, public health, multiple chronic conditions, artificial intelligence (AI)

Multimorbidity—the coexistence of two or more long-term conditions within an individual—has become a defining challenge in population health. Prevalence is increasing globally, particularly among older adults and socioeconomically disadvantaged groups. Multimorbidity is associated with diminished quality of life, high service utilisation, fragmented care, and widening health inequalities. Public health urgently needs scalable, ethical, and interpretable solutions to understand, monitor, and address its complexity. Yet, the tools traditionally used in public health research and planning—such as regression-based models and manual cohort curation—are fundamentally limited in addressing the scale, dynamism, and heterogeneity that characterise multimorbidity today. In this context, artificial intelligence (AI) offers a necessary evolution in our approach to health data science—one that can meet the demands of increasingly complex datasets and enable proactive, data-driven public health action [1]. AI allows the integration of diverse data sources, from clinical records to social determinants, enabling analyses that uncover hidden associations and inform targeted interventions at population scale.

In our own work, we encountered one of the most foundational barriers to large-scale multimorbidity research: dataset curation. When using the English Longitudinal Study of Ageing (ELSA) [2]—a cohort with over 94,000 variables across multiple waves—manual selection and harmonisation of variables proved slow, inconsistent, and dependent on expert knowledge. Each wave introduced subtle definitional differences, requiring careful interpretation that often takes months to complete. To address this, we developed and evaluated two AI-driven pipelines. A semantic search pipeline using natural language processing achieved a high AUC (0.899) (AUC - Area Under the Receiver Operating Characteristic Curve: a measure of how well a model can distinguish between classes; higher AUC means better discrimination), effectively identifying relevant variables in response to domain-specific queries [3]. This enabled us to retrieve semantically related variables, even when terminology differed across datasets. A second semantic clustering pipeline, although with a more modest V-measure (0.237), which is a score that checks how well computer-made groups match the real groups by balancing homogeneity (each group is consistent) and completeness (all similar items are kept together) [4]. This clustering approach demonstrated the feasibility of AI-assisted harmonisation across study waves [5]. Most critically, these tools achieved over a 100-fold speed increase compared to manual curation, making previously unmanageable datasets usable for public health analysis. This step-change in efficiency has major implications for longitudinal and population studies, allowing more timely insights and cross-cohort comparisons.

In another application, we used the Clinical Practice Research Datalink (CPRD) to explore the social care needs of individuals with multimorbidity across a 20-year period, covering nearly 280,000 individuals. Traditional subgrouping methods like latent class analysis were infeasible given the scale—over 500,000 data points from routine clinical care. We instead used Mini-Batch K-means, which is a clustering variant of the K-Means method, using small, randomly selected subsets of data rather than the entire dataset that significantly reduces clustering time comparable with traditional K-Means [6]. Evaluated using silhouette scores (average >0.9), the resulting 15 clusters represented robust and distinct care need profiles, spanning over 2 million person-years. These insights revealed natural groupings of patients based not only on their conditions, but also on associated care requirements—information vital for commissioners, integrated care systems, and public health teams. This AI-enabled approach moves beyond descriptive statistics and towards actionable segmentation: understanding who needs what type of care, when, and why. In public health terms, this supports resource targeting, proactive outreach, and equitable service design [7]. The capacity to integrate medical, demographic, and social data at such scale offers an unprecedented opportunity to anticipate demand and plan services in a way that is equitable and cost-effective.

AI presents transformative opportunities for the public health response to multimorbidity: Surveillance: Clustering and predictive models can identify emerging multimorbidity patterns in ageing or marginalised populations [8]. Precision prevention: Stratifying populations by condition combinations, medication profiles, or social risk factors allows for better-targeted interventions and more effective use of resources. System planning: AI can inform where to locate multidisciplinary teams, community care hubs, or social prescribing schemes by anticipating local need [9]. These applications signal a paradigm shift in how population health challenges can be addressed, moving toward proactive, predictive, and personalised approaches.

The challenge is not technical capability—it is uptake, governance, and public trust. Despite the promise, AI in public health brings important risks that must be proactively managed. Algorithmic bias remains a major concern. If trained on incomplete or non-representative data, AI models may entrench inequalities in care access and quality [10]. Similarly, black-box tools lacking explainability may be misapplied or misunderstood in community settings. Transparency, fairness, and explainability must therefore be foundational in all AI development and deployment. Models should be evaluated across demographic subgroups, incorporate principles of algorithmic accountability, and be designed with public input and oversight. Additionally, capacity building is essential. Public health professionals need the skills and governance frameworks to critically appraise AI outputs, ask the right questions, and use these tools effectively.

Multimorbidity challenges nearly every assumption of traditional public health surveillance and intervention design. Conditions do not act independently. Social context cannot be “adjusted for” and ignored. Data volume and complexity now exceed manual analytic capacity. AI—used responsibly—can help us move from descriptive to predictive, from reactive to preventive, and from fragmented to integrated care planning. In our own work, we have seen how AI accelerates access to meaningful patterns in large and messy datasets. But success depends not only on innovation, but also on transparency, ethics, and alignment with core public health values. As the prevalence and impact of multimorbidity grow, so too must our analytical ambition. AI is not a luxury—it is a public health imperative.
